# Long-range quantum entanglement in dielectric mu-near-zero metamaterials

**DOI:** 10.1038/s41377-025-01994-9

**Published:** 2025-09-03

**Authors:** Olivia Mello, Larissa Vertchenko, Seth Nelson, Adrien Debacq, Durdu Guney, Eric Mazur, Michaël Lobet

**Affiliations:** 1 John A. Paulson School of Engineering and Applied Sciences, Harvard University, 9 Oxford Street, Cambridge, MA 02138 USA; 2 Sparrow Quantum, 2000 Copenhagen, Denmark; 3 Physics Department, Michigan Technological University, Houghton, MI USA; 4 Department of Physics and Namur Institute of Structured Materials, University of Namur, Rue de Bruxelles 51, 5000 Namur, Belgium; 5 Electrical & Computer Engineering Department, Michigan Technological University, Houghton, MI USA

**Keywords:** Metamaterials, Optics and photonics

## Abstract

Entanglement is paramount in quantum information processing. Many quantum systems suffer from spatial decoherence in distances over a wavelength and cannot be sustained over short time periods due to dissipation. However, long range solutions are required for the development of quantum information processing on chip. Photonic reservoirs mediating the interactions between qubits and their environment are suggested. Recent research takes advantage of extended wavelength inside near-zero refractive index media to solve the long-range problem along with less sensitivity on the position of quantum emitters. However, those recent proposals use plasmonic epsilon near-zero waveguides that are intrinsically lossy. Here, we propose a fully dielectric platform, compatible with the Nitrogen Vacancy (NV) diamond centers on-chip technology, to drastically improve the range of entanglement over 17 free-space wavelengths, or approximatively 12.5 µm, using mu near-zero metamaterials. We evaluate transient and steady state concurrence demonstrating an order of magnitude enhancement compared to previous works. This is, to the best of our knowledge, the first time that such a long distance is reported using this strategy. Moreover, value of the zero time delay second order correlation function ${g}_{12}^{(2)}(0)$ are provided, showing antibunching signature correlated with a high degree of concurrence.

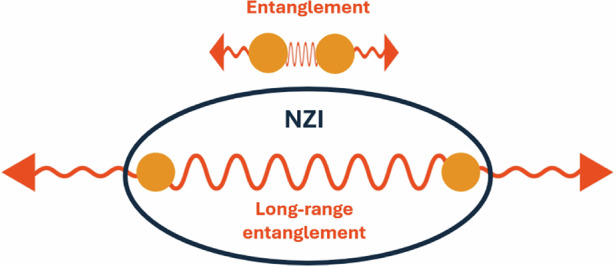

## Abstract (web summary)

We propose a mu-near-zero index platform, a kind of near-zero refractive index materials, that enable long-range entanglement between on-chip quantum emitters. Transient and steady state concurrence demonstrating an order of magnitude enhancement compared to previous works.

## Introduction

A principal topic in quantum information processes is generating and maintaining entanglement^1^. Entanglement, which is defined as the non-separability of quantum states, is important for quantum teleportation, quantum metrology, quantum communication, and quantum computing. First demonstrated in optical and atomic/ionic systems^1,2^, entanglement has now been demonstrated in a variety of solid-state systems, including Josephson junctions^3^, the spin of quantum dots^4^, and in single photon emission from silicon carbide^5^. However, in solid-state on-chip systems, entanglement is observed over distances within only one spatial wavelength or not much greater^6^. In order to have information transfer over long distances, entanglement and interactions between qubits must be maintained over large spatial separations, avoiding decoherence from coupling of the quantum emitters and their environment^7^.

It has been shown that while typically spontaneous emission is a form of decoherence in most entanglement regimes, because it is a source of dissipation into the environment, this dissipation can also be a source of entanglement generation^8,9^ for two-level systems interacting in a bosonic field. This is particularly relevant for the case of cooperative enhancement of spontaneous emission via Dicke states^10^. Dicke states correspond to the symmetric and antisymmetric linear combinations of the ground ${|g}\rangle$ and excited states ${|e}\rangle$ for a collection of two-level systems. For a set of two qubits, the symmetric superradiant $|+\rangle =({|e}\rangle {|g}\rangle +{|g}\rangle {|e}\rangle )/\sqrt{2}$ and antisymmetric subradiant $|-\rangle =({|e}\rangle {|g}\rangle -{|g}\rangle {|e}\rangle )/\sqrt{2}$ Dicke states are the maximally entangled states in the system, as we cannot factor them. As cooperative spontaneous emission mediates interactions with these states, it is one means of entanglement generation for such systems.

There have been multiple proposals for long-distance entanglement, such as the Duan–Lukin–Cirac–Zoller (DLCZ) protocol between two distant cold atom cavity ensembles^11^. Long-distance heralded entanglement with optical photons has been experimentally demonstrated with distant quantum dot hole spins^12^ and with electron spins in diamond nitrogen vacancy centers^13^. Such protocols are difficult to attain on an on-chip system because they require, for example, complicated entanglement preparation protocols involving entanglement swapping^14^ and a separate intermediary location between the two qubits. The cooperative quantum optical effects that generate entanglement on a mesoscopic (on-chip) scale rapidly depreciate after length scales greater than one interaction wavelength (${kr} < 1$ for having cooperative effects, with $k$ the wavenumber, $r$ the position and $\lambda$ the wavelength inside the material). There have been several proposals to use photonic reservoirs to mediate the interactions between qubits as well as their environment^15^.

In that context, the development of near-zero refractive index (NZI) photonics is of prime interest^16–18^. Indeed, inside such NZI media, the effective wavelength $\lambda =\frac{{\lambda }_{0}}{n}$ stretches to infinity while the wavevector goes to zero^19^, therefore quantum emitters are able to cooperate because of enhanced coherence length. As the refractive index is defined by $n=\pm \sqrt{\varepsilon \mu }$, the NZI state can be reached by 3 different ways: either the electric permittivity $\varepsilon$ is close to zero—the epsilon near-zero (ENZ) category—the magnetic permeability $\mu$ is close to zero—the mu near-zero (MNZ) category—or both $\varepsilon$ and $\mu$ are close to zero—the epsilon and mu near-zero (EMNZ) category^20^.

Quantum entanglement mediated by ENZ reservoirs^8,21–24^ has been recently discussed as a system conferring both local field enhancement and constructive interference due to the relaxed phase matching conditions^25^ at the ENZ frequency. Those are plasmonic waveguide systems that operate at the cutoff frequency to generate the ENZ behavior. Despite great interest, those plasmonic systems are intrinsically lossy. To tackle this problem, based on all-dielectric NZI metamaterials^18,26–28^, we previously designed a diamond ENZ metamaterial platform that theoretically and numerically showed superradiant decay rate enhancement over distances greater than 13 times the free-space wavelength^29^. A power enhancement of three orders of magnitude higher than an incoherent array of emitters in bulk diamond was observed, corresponding to an ${N}^{2}$ scaling with the number of emitters as a characteristic of superradiance. While interesting from a low-loss point of view, this 2D ENZ platform presents a natively high impedance $Z=\sqrt{\frac{\mu }{\varepsilon }}$ as we approach the NZI frequency, hence hindering in and out-coupling with the structure. Nevertheless, as recently shown, both the category of NZI and the spatial dimensions of the photonic media have a drastic impact on fundamental radiative processes such as spontaneous emission^30^. Therefore, investigating other NZI categories could potentially solve this impedance problem.

Here, we demonstrate how planar MNZ metamaterials can significantly improve the range of entanglement over more than 10 free-space wavelengths. This MNZ design has a transverse electric (TE) polarization with a finite impedance. For systems of entangled qubits in emitter-based systems on-chip, our system represents a significant improvement in entanglement over distance between the emitters. Additionally, the method of entanglement generation in this system is comparatively quite simple, based on the cooperative spontaneous emission between emitters that we appreciate due to the significantly reduced phase advance in near-zero refractive index materials.

In our theoretical model, we utilize the calculated^29^ coupling coefficients and decay rates in the Lindblad master equation to describe the quantum dynamics of a two-qubit system through its density matrix. We provide the general coupled differential equations for the density matrix elements of the system in the presence of pumps to describe the full quantum dynamics of the two-qubit system under different initial conditions. These equations can be readily used to implement various quantum tasks such as entanglement generation, quantum logic gates, quantum teleportation, and other two-qubit quantum operations in metamaterials and photonic crystals akin to^31,32^.

## Results

### Theoretical framework of entanglement in two-level systems

**Fig. 1:**
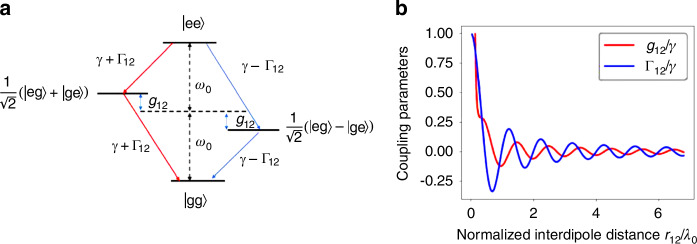
Two two-level systems and coupling parameters. **a** System of two two‐level atoms with symmetric and antisymmetric states $|+\rangle$ and $|-\rangle$ with cooperative decay rates $\gamma \pm {\varGamma }_{12}$ and cooperative frequency level shifts ${g}_{12}$. **b** Plots of the normalized cooperative decay rate ${\varGamma }_{12}$ and the dipole‐dipole couplings ${g}_{12}$ in free space, as a function of the normalized interdipole distance

The two-level atomic system considered here is represented in Fig. 1a with the corresponding

Hamiltonian describing the coherent part of the dynamics between two emitters (Fig. 1a) being

1 $$H=\mathop{\sum}\limits_{i}{{\hslash }}({\omega }_{0}+{g}_{{ii}}){\sigma }_{i}^{\dagger }{\sigma }_{i}+\mathop{\sum}\limits_{i\ne j}{{\hslash }}{g}_{{ij}}{\sigma }_{i}^{\dagger }{\sigma }_{j}$$

where ${g}_{{ii}}$ represents the photonic Lamb shift^22,33^ generated as a result of self-interaction with a single emitter. The Lamb shift arises when the bound electrons interact with fluctuations in the surrounding vacuum field in the medium. This shift is typically incorporated into the transition frequency term of any chosen emitter and thus does not affect our calculations here. ${\sigma }_{i,j}$ are the ladder operators between the excited states $|{e}_{\mathrm{1,2}}\rangle$ and the ground states $|{g}_{\mathrm{1,2}}\rangle$ for emitters 1 and 2. The coherent dipole–dipole coupling term ${g}_{{ij}}$ represents interactions that result in a frequency shift between emitters. Figure 1a illustrates how the dipole–dipole coupling term affects the frequency shift in the symmetric and antisymmetric states in the system of two two-level atoms. In resonant structures, we can calculate this term as the real part of the Green’s tensor $\mathop{G}\limits^{\leftrightarrow}({r}_{i},{r}_{j},{\omega }_{j})$^22,29,34^,

2 $${g}_{{ij}}=\frac{2{\omega }_{j}^{2}}{\hslash {\varepsilon }_{0}{c}^{2}}{d}_{i}{\mathrm{Re}}\left[\mathop{G}\limits^{\leftrightarrow}\left({r}_{i},{r}_{j},{\omega }_{j}\right)\right]{d}_{j}^{* }$$

where ${{\boldsymbol{d}}}_{i,j}$ are the dipole moments of the *i*th and *j*th emitters at positions ${{\boldsymbol{r}}}_{i}$ and ${{\boldsymbol{r}}}_{j}$ for a frequency ${\omega }_{j}$.

The collective spontaneous emission rates ${\gamma }_{{ij}}$, defined as ${\varGamma }_{12}$ for the two-atom system (Fig. 1a), arise from coupling between the atoms through the vacuum field. The cooperative decay rates ${\gamma }_{{ij}}={\varGamma }_{12}$ and the single-atom spontaneous decay rate ${\gamma }_{{ii}}=\gamma$ are determined by the imaginary component of the Green’s function:

3 $${\gamma }_{{ij}}=\frac{2{\omega }_{j}^{2}}{\hslash {\varepsilon }_{0}{c}^{2}}{\boldsymbol{d}}_{i}{Im}\left[\mathop{G}\limits^{\leftrightarrow}({r}_{i},{r}_{j},{\omega }_{j})\right]{d}_{j}^{* }$$

It should be noted that the cooperative decay rate ${\gamma }_{12}={\varGamma }_{12}$ and single-atom spontaneous decay rate ${\gamma }_{{ii}}$ factor in when calculating the density matrix, as they are the dissipative components in the Lindblad master equation^34,35^:

4 $$\frac{\partial \rho }{\partial t}=\frac{1}{i\hslash }\left[H,\rho \right]-\frac{1}{2}\mathop{\sum}\limits_{i,j}^{2}{\gamma }_{i,j}\left(\rho {\sigma }_{i}^{\dagger }{\sigma }_{j}+{\sigma }_{i}^{\dagger }{\sigma }_{i}\rho -2{\sigma }_{i}\rho {\sigma }_{j}^{\dagger }\right)$$

with ${\sigma }_{i}=|{g}_{i}\rangle \langle {e}_{i}|$ is the ladder operators for the *i*th emitter.

In free space or inside a homogeneous medium with a wavevector $k$, the cooperative decay rate and the dipole–dipole coupling can be evaluated analytically^36,37^ as

5 $${\gamma }_{i,j}=\frac{3\gamma }{4}\left(\left[1-{\left(\bar{\boldsymbol{d}}\cdot {\bar{\boldsymbol{r}}}_{\boldsymbol{ij}}\right)}^{2}\right]\right)\frac{\sin \left({kr}\right)}{{kr}}+\left(\left[1-3{\left(\bar{\boldsymbol{d}}\cdot {\bar{\boldsymbol{r}}}_{\boldsymbol{ij}}\right)}^{2}\right]\right)\left[\frac{\cos \left({kr}\right)}{{\left({kr}\right)}^{2}}-\frac{\sin \left({kr}\right)}{{\left({kr}\right)}^{3}}\right]$$

6 $${g}_{i,j}=\frac{3\gamma }{4}\left(-\left[1-{\left({\bar{\boldsymbol{d}}}\cdot {\bar{\boldsymbol{r}}}_{\boldsymbol{ij}}\right)}^{2}\right]\right)\frac{\cos \left({kr}\right)}{{kr}}+\left(\left[1-3{\left(\bar{\boldsymbol{d}}\cdot {\bar{\boldsymbol{r}}}_{\boldsymbol{ij}}\right)}^{2}\right]\right)\left[\frac{\sin \left({kr}\right)}{{\left({kr}\right)}^{2}}+\frac{\cos \left({kr}\right)}{{\left({kr}\right)}^{3}}\right]$$

where *γ* is the free space spontaneous emission rate and $\bar{{\boldsymbol{d}}}$ and $\bar{{\boldsymbol{r}}}$ are unit vectors along the atomic transition dipole moments and the vector ${\boldsymbol{r}}_{{ij}}={\boldsymbol{r}}_{j}-{\boldsymbol{r}}_{i}$, respectively.

Figure 1b shows the plots for the cooperative dissipative coupling for two dipoles ${\varGamma }_{12}$ and the coherent dipole–dipole coupling term ${g}_{12}$ over a normalized interdipole separation in free space. As the separation between the two dipoles exceeds one wavelength for the free space case, both the cooperative decay and dipole–dipole coupling terms rapidly degrade. We will observe how these two terms affect entanglement and how we can engineer them to sustain entanglement over extents greater than 10 free-space wavelengths or more.

Introduced by Wooters^38^, concurrence helps to characterize the extent to which two emitters are entangled with one another. The concurrence $C$ is defined as

7 $$C=\max \left(0,\sqrt{{\lambda }_{1}}-\sqrt{{\lambda }_{2}}-\sqrt{{\lambda }_{3}}-\sqrt{{\lambda }_{4}}\right)$$

where ${\lambda }_{i}$ are the eigenvalues of the matrix $\rho \widetilde{\rho }$ in descending order and $\widetilde{\rho }=\left({\sigma }_{y}\otimes {\sigma }_{y}\right){\rho }^{* }({\sigma }_{y}\otimes {\sigma }_{y})$ and ${\sigma }_{y}$ is the 2-by-2 Pauli matrix. Therefore, for completely unentangled states, *C* = 0 and for a maximally entangled system, *C* = 1. To calculate the concurrence, we must solve the time-dependent density matrix components $\rho (t)$ with the Lindblad master equation (Eq. (4)). We solve it in the Dicke basis: $|0\rangle =|{g}_{1}\rangle |{g}_{2}\rangle ,{|}3\rangle ={|e}1\rangle {|e}2\rangle ,|+\rangle =(|{e}_{1}\rangle |{g}_{2}\rangle +|{g}_{1}\rangle |{e}_{2}\rangle)/\sqrt{2}\,and\,{|}-\rangle =(|{e}_{1}\rangle |{g}_{2}\rangle -|{g}_{1}\rangle |{e}_{2}\rangle)/\sqrt{2}.$

Up until this point, we have considered the emitter decay and dipole–dipole coupling due to its interaction with the electromagnetic field of the photonic reservoir. However, depending on the emitter, there can be other non-radiative channels for decay, known as dephasing. The master equation can additionally concern itself with dephasing $\gamma {\prime}$^8,35,39^. The contribution of dephasing to the dynamics of the master equation is

8 $$L\rho =\mathop{\sum}\limits_{i=1,2}\frac{{\gamma }^{{\prime} }}{2}\left[{2\sigma }_{i}^{\dagger }{\sigma }_{i}\rho {\sigma }_{i}{\sigma }_{i}^{\dagger }-{{\sigma }_{i}\sigma }_{i}^{\dagger }{\sigma }_{i}^{\dagger }{\sigma }_{i}\rho -\rho {{\sigma }_{i}\sigma }_{i}^{\dagger }{\sigma }_{i}^{\dagger }{\sigma }_{i}\right]$$

For example, in the silicon vacancy in diamond, several sources are responsible for dephasing. A strain-induced increase of orbital splitting to an energy at which phonon population is one contribution^40^. Phonon excitations in the diamond lattice from the lower to the upper orbital branches in the ground-state manifold are the largest contribution. Dephasing in the silicon vacancy center is highly dependent on temperature, making dilution refrigeration^40^ an often important aspect to measurement. For the sake of our calculations, we assume a system at low temperatures such that the dephasing terms typically present in the master equation are small. With these considerations, we can calculate the terms $\partial {\rho }_{{ij}}/\partial t$ in the Dicke basis.

To solve this set of equations, we assume a single initial excitation where we prepare the initial unentangled state $|{e}_{1},\,{g}_{2}\rangle =\frac{1}{\sqrt{2}}(|+\rangle +|-\rangle )$. This gives us the initial conditions for the density matrix at $t=0,{\rho }_{++}(0)={\rho }_{--}(0)={\rho }_{+-}(0)={\rho }_{-+}(0)=1/2$, and ${\rho }_{33}(0)={\rho }_{00}(0)=0$. This greatly simplifies our set of differential equations, and the time-dependent solution for the nonzero elements of the density matrix becomes

9 $$\left\{\begin{array}{c}{\rho }_{++}\left(t\right)=\frac{1}{2}{{\rm {e}}}^{-\left(\gamma +{\Gamma }_{12}\right)t}\\ {\rho }_{--}\left(t\right)=\frac{1}{2}{{\rm {e}}}^{-\left(\gamma -{\Gamma }_{12}\right)t}\\ {\rho }_{+-}\left(t\right)=\frac{1}{2}{{\rm {e}}}^{-\left(\gamma -2i{g}_{12}\right)t}\\ {\rho }_{-+}\left(t\right)=\frac{1}{2}{{\rm {e}}}^{-\left(\gamma +2i{g}_{12}\right)t}\end{array}\right.$$

If the density matrix of this system only contains the four above elements, the expression of the concurrence can be significantly simplified^8^ to

10 $$C\left(t\right)=\sqrt{{\left({\rho }_{++}-{\rho }_{--}\right)}^{2}+4{Im}{\left({\rho }_{+-}\right)}^{2}}$$

With the density matrix solutions above, the expression for the concurrence reduces to

11 $$C=\frac{1}{2}\sqrt{{\left({{\rm {e}}}^{-\left(\gamma +{\varGamma }_{12}\right)t}-{{\rm {e}}}^{-\left(\gamma -{\varGamma }_{12}\right)t}\right)}^{2}+4{{\rm {e}}}^{-2\gamma t}{\sin }^{2}\left(2{g}_{12}t\right)}$$

For a configuration of two emitters in free space, we observe a transient concurrence that rapidly degrades as soon as the two emitters are separated as little as half a wavelength. We can observe from the expression of the concurrence that for ideal concurrence values, ${\varGamma }_{12}/\gamma =1$ for large inter-emitter separations and ${\varGamma }_{12}\gg {g}_{12}$. As we demonstrated in previous work^29^, near-zero-index metamaterials, particularly ENZ metamaterials, exhibit a strong spatial enhancement in cooperative decay rate. Here, we demonstrate that MNZ metamaterials display cooperative enhancement in the decay terms ${\varGamma }_{{ij}}$ over large spatial extents. This is because, while the retrieved effective permittivity ${\varepsilon }_{r}$ is a nonzero value, the effective index ${n}_{{\rm{eff}}}$ still approaches the near-zero limit at the wavelength in which ${\mu }_{r}$ crosses zero.

### Design of the MNZ metamaterial

Recently, the development of NZI photonics showed a range of particularly interesting effects for light and thermal emission, nonlinear optics, sensing applications, and time-varying photonics^20^. This includes, for example, applications such as supercoupling^16^, cloaking^19,41,42^, crosstalk prohibition^43^, photonic doping^44^, or impurity-immunity^45^.

**Fig. 2:**
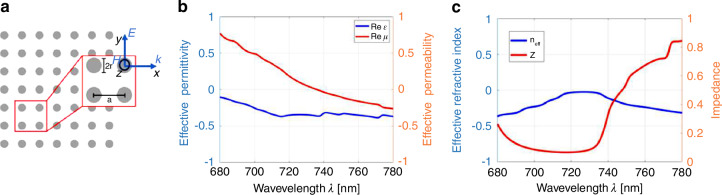
Parameter retrieval. **a** Schematic of the 2D square metamaterial lattice with a pitch $a$ and radius $r$. **b** Effective parameters ${\varepsilon }_{r}$ and ${\mu }_{r}$ with a $\mu$ zero crossing at 737 nm. **c** The retrieved effective refractive index ${n}_{{{\rm {eff}}}}$ and impedance $Z$

We start by numerically designing a planar MNZ metamaterial composed of a square lattice structure of period $a=505\,{{\rm {nm}}}$ with a pillar radius $r=115\,{{\rm {nm}}}$ (Fig. 2a). The quantum emitter will be based on silicon vacancies (SiVs) in diamond, with a corresponding zero-phonon line at 737 nm. Excitation comes from a fundamental transverse electric (TE) mode source. The MNZ mode is TE polarized, with the field ${H}_{z}$ polarized out of plane.

Although the period $a$ in this case is not much smaller than the operating wavelength ${\lambda }_{0}=737\,{{\rm {nm}}}$ in vacuum, it should be noted that the effective wavelength $\lambda ={\lambda }_{0}/{n}_{{\rm{eff}}}$ inside the MNZ metamaterial is bigger than $a$ once the effective refractive index is close to zero. Therefore, we follow the formalism of the homogenization criteria in the limit of short optical wavelengths^46^. The effective refractive index ${n}_{{\rm{eff}}}$ can be retrieved by recording the average change in phase between each pillar in the simulation region, while the impedance $Z$ and the effective constitutive parameters ${\varepsilon }_{{\rm {r}}}$ and ${\mu }_{{\rm {r}}}$ are retrieved by calculating the transfer matrix^29,47–50^ using the transmitted and reflected fields recorded by the monitors near the boundaries of the metamaterial.

Figure 2b shows that, as imposed by design, the real part of the effective permeability crosses zero at 737 nm, the zero-phonon line of the silicon vacancy center, while the effective permittivity ${\varepsilon }_{{\rm {r}}}=-0.36$. Figure 2c shows the corresponding minimum absolute value of the effective phase index ${n}_{{\rm{eff}}}=-0.03$ at the wavelength of the $\mu$ zero crossing. This corresponds to a low value of impedance^27^, as $Z=\sqrt{\frac{\mu }{\varepsilon }}=0.2$. The reason that the value of ${n}_{{\rm{eff}}}$ never fully crosses zero is that the retrieved optical parameters ${\varepsilon }_{{\rm {r}}}$ and ${\mu }_{{\rm {r}}}$ have small imaginary components (see Figs. S1 and S2 in SI for ${{\rm {Im}}}\left({\varepsilon }_{{\rm {r}}}\right.$), ${{\rm {Im}}}\left({\mu }_{{\rm {r}}}\right.$) and the impact on ${{\rm {Im}}}\left({n}_{{\rm{eff}}}\right)$). This further indicates low losses from these imaginary contributions.

Furthermore, to double-check the consistency of the retrieved parameters using the parameter retrieval methods, we calculated the effective index by averaging the phase advance between each pillar for 10 periods of the metamaterial using Ansys Lumerical FDTD. This agreement (see Fig. S3 in SI) confirms the validity of the photonic crystal as an MNZ metamaterial structure.

**Fig. 3:**
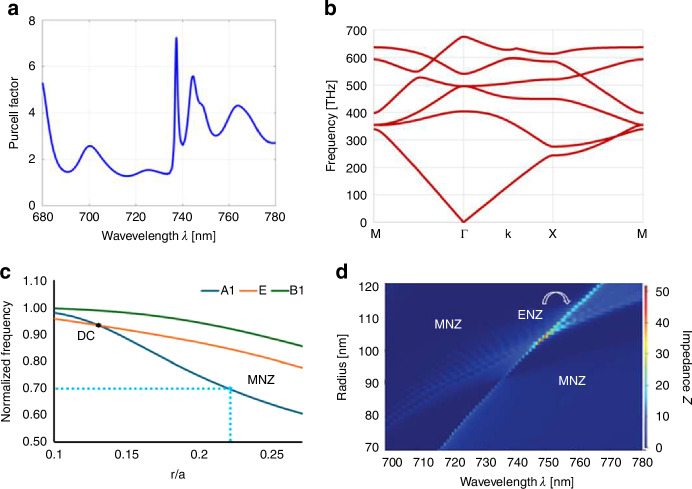
Photonic band structure, Purcell factor and impedance calculations. **a** Spectrum of the Purcell factor in the MNZ structure, with a peak near 737 nm. **b** Band structure of the 2D square lattice of dielectric pillars ($n=2.4064$) with a pitch $a=505\,{{\rm {nm}}}$ and radius *r* = 115 nm. **c** Plot of the eigenfrequency at the $\varGamma$-point of the A1 (monopolar) mode, the two degenerate E (dipolar) modes, and the B1 (quadrupolar) mode as we change the radius from the EMNZ design value (Dirac cone). **d** Plot of the impedance spectrum with various radius showing the MNZ (blue) and ENZ (bright diagonal) regions

The low impedance justifies the choice of an MNZ metamaterial compared to an ENZ metamaterial^29^. The ENZ designs^19,30^, while demonstrating a high Purcell enhancement relative to the large mode volumes that near-zero-index modes occupy, cannot couple effectively in or out of plane. This lack of efficient coupling is experimentally detrimental. In contrast, the MNZ design gives a lower but nonzero impedance, making it much easier to couple radiation in or out of the material both in or out of plane (see discussion in SI for quantification and distinction with an EMNZ case). This coupling possibility is a particular advantage when working with low-index materials such as diamond, as they require either suspended or angle-etched waveguides to efficiently couple out of the metamaterial without radiation leaking into the substrate. A modest Purcell enhancement at the $\mu$ near-zero crossing at 737 nm is still present $({F}_{{\rm {P}}}=7)$ as indicated in Fig. 3a, because we are still operating at a shallow parabolic band edge at the center of the Brillouin zone Γ (Fig. 3b).

The MNZ structure is derived from an EMNZ structure with a triply degenerate Dirac cone dispersion^17,18,26,41,51^ coming from the degeneracy of the monopolar $$A1$$ mode and the doubly degenerated dipolar $$E$$ modes^51^. The original 2D EMNZ diamond metamaterial structure has a pitch $$a=684\,{{\rm {nm}}}$$ and radius $$r=87\,{{\rm {nm}}}$$. Figure 3c shows how detuning the radius and re-scaling the pitch, we can break the degeneracy of these three bands (Dirac cone occurring at the normalized frequency $$0.92$$ for $$r/a=0.13$$) and again operate near a band edge after breaking the degeneracy of these modes (MNZ mode occurring at the normalized frequency $$0.7$$ for $$r/a=0.22)$$. In Fig. 3d, we similarly see how breaking the degeneracy of the modes by either increasing or decreasing the radius controls the impedance, as we are changing the values of $$\varepsilon$$ and $$\mu$$ relative to one another. Near the spike of impedance for shrunken pillars, we see a theoretical ENZ behavior (bright dots in Fig. 3d). However, in our simulations, we do not see any local or cooperative enhancement at this point because of the existence of an additional band, the flat band, at this point. As described in detail in refs. ^41,51^, the flat band has propagation that is longitudinal at that point, which causes destructive interference in the fields. It is specifically due to the TE-polarization with the $${H}_{z}$$ monopole mode that we observe MNZ behavior instead of ENZ, as is the case for the TM-polarized ENZ structure in ref. ^29^.

**Fig. 4:**
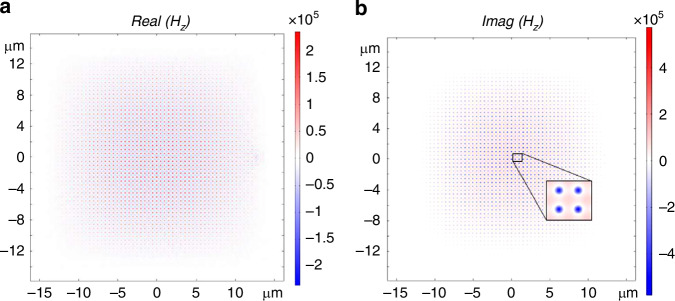
Magnetic fields calculations. **a** The real and **b** imaginary components of the ${H}_{z}$ field for the MNZ monopole mode

Figure 4 shows the real and imaginary components of the MNZ TE modes for a 51 × 51 pitch design extending over ~12 µm. Both the real and imaginary components of the field extend nearly throughout the metamaterial, demonstrating ”supermode”-like behavior where the metamaterial generates larger-scale fundamental Fabry–Pérot cavity mode^22,29,52^. Figure 4b demonstrates that the MNZ mode is the monopolar $A1$ mode with a TE-polarization that corresponds to the magnetic resonance that generates the $\mu$ near-zero crossing. The imaginary component of the ${H}_{z}$ field, which is proportional to ${\varGamma }_{12}$, is roughly three times larger in magnitude than the real part of ${H}_{z}$, which is proportional to the dipole–dipole coupling ${g}_{12}$. Having a ${\varGamma }_{12}\gg {g}_{12}$ is ideal for attaining a large transient concurrence as determined by the Hamiltonian of our system. We further investigate the long-range entanglement properties of the designed MNZ metamarials hereafter.

### Entanglement in the MNZ MM

Having the MNZ metamaterial designed allows us to calculate dipole–dipole coupling ${g}_{12}$ (Eq. (2)) and the cooperative decay rate ${\gamma }_{12}$ (Eq. (3)). We use full-wave numerical simulations (COMSOL Multiphysics) by sweeping a magnetic dipole source from the origin to 25 pitches away along the $x$-direction. As the dipole moves from pillar to pillar along the $X-M$ direction, the real and imaginary components of the ${H}_{z}$ field from the TE monopole mode are recorded. As observed in Fig. 4, the fields are radially isotropic upwards of 8 μm, therefore transferring that isotropic property to both cooperative enhancement and dipole–dipole coupling.

**Fig. 5:**
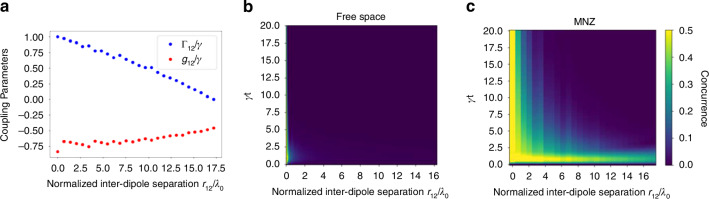
Coupling parameters and transient concurrence in MNZ metamaterial. **a** Normalized cooperative decay coupling ${\varGamma }_{12}$ and dipole–dipole coupling ${g}_{12}$ calculated from moving the dipole source across MNZ structure. **b** Plot of the transient concurrence as a function of the normalized interdipole separation for free space and **c** the MNZ metamaterial

Figure 5a demonstrates the cooperative enhancement ${\varGamma }_{12}/\gamma$ and dipole–dipole coupling ${g}_{12}/\gamma$, both normalized by the spontaneous decay rate (calculated from pillar to pillar) in the MNZ metamaterial design. For the MNZ material, we use magnetic dipole sources with the dipole moment aligned along the length of the pillars. Using such a source in simulations corresponds to the TE-polarization of the MNZ material with the ${H}_{z}$ mode aligned along the axis of the pillars. By comparing with Fig. 1b (free space), one can observe extended cooperative enhancement for over an order of magnitude longer inter-emitter separations inside the MNZ metamaterial. This enhanced cooperative enhancement ${\varGamma }_{12}/\gamma > 0$ is sustained for over 17 free-space wavelengths (${\lambda }_{0}=737\,{{\rm {nm}}}$), so about 12.5 µm, which is one order of magnitude higher than in the rolled-up or rectangular ENZ waveguides ($1.5r/{\lambda }_{0})$^23^. Moreover, the coherent dipole–dipole coupling terms ${g}_{12}/\gamma$ are smaller than those of the free-space dipole–dipole coupling terms, which diverge for short inter-emitter separations. ${g}_{12}/\gamma$ has the same trend as the ENZ waveguides described in ref. ^24^ with more negative values over an extended range.

With the optical coupling parameters ${\varGamma }_{12}$ and ${g}_{12}$ at hand, we can calculate the transient concurrence from Eq. (11). Figure 5b and c show the plot of transient concurrence over a normalized time $\gamma t$ and over a wavelength-normalized inter-emitter separation for free space and in the MNZ structure. Note that in both figures the concurrence at $\gamma t=0$ is $C(0)=0$ as there has yet to be a spontaneous decay event to induce entanglement via cooperative decay through the symmetric superradiant state.

Shortly after a spontaneous decay event, a high concurrence is maintained throughout the MNZ structure (Fig. 5c), in contrast to free space, where the concurrence goes to zero almost immediately. There is robust entanglement shortly after $\gamma t=0$ that, which persists for spatial separations of over 20 times the extent of the case of the concurrence in a vacuum system. This temporal variation is similar to previous work^23^. The concurrence remains high, upwards of 0.35, for even 17 wavelengths, or roughly 12.5 µm. This distance of high concurrence entanglement is nearly an order of magnitude longer than the one in the ENZ plasmonic waveguides^21,23^. Not only is there a drastic spatial improvement of entanglement in the MNZ structure compared to in free space (Fig. 5b), but there is also a significant temporal enhancement in the concurrence as it decays with time.

As the concurrence is transient, after a long period of time, the system needs to be sustained by an external source to prolong the entanglement. To remedy this, we can apply an external pump source to both qubits to observe steady-state behavior. For a pump potential

12 $$V=-\mathop{\sum}\limits_{i=1}^{2}\hslash \left({\varOmega }_{i}{{\rm {e}}}^{-i{\varDelta }_{i}t}{\sigma }_{i}^{\dagger }+{\varOmega }_{i}^{* }{{\rm {e}}}^{i{\varDelta }_{i}t}{\sigma }_{i}\right)$$

we add a term $\frac{1}{i\hslash }[V,\rho ]$ to the right-hand side of the master equation in Eq. (4). The effective Rabi terms are ${\varOmega }_{i}$ for the *i*th emitter in the MNZ medium. The parameter ${\varDelta }_{i}=\omega -{\omega }_{{\rm {p}}}$ is the pump detuning term for a pump frequency ${\omega }_{{\rm {p}}}$. For the sake of the following calculations, we assume that the pump operates at the same frequency as the resonant frequency of the qubits. This potential term significantly complicates the equations for the time evolution of the density matrix $\rho (t)$. It is preferable to solve this set of equations in this basis of $|0\rangle ={|g}1,{g}2\rangle ,{|}1\rangle ={|g}1,{e}2\rangle ,{|}2\rangle ={|e}1,{g}2\rangle ,{and\; |}3\rangle ={|e}1,{e}2\rangle$. Using the standard basis and the potential term in Eq. (4) and assuming zero detuning terms ${\varDelta }_{i}=0$, we obtain a system of 16 equations (see SI).

**Fig. 6:**
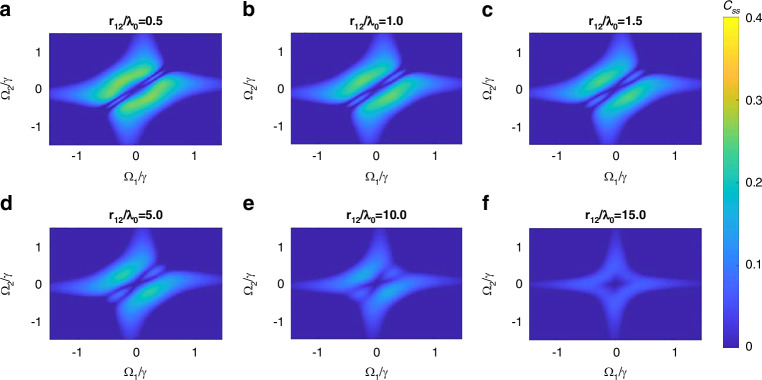
Steady-state concurrence in MNZ metamaterial. Steady-state concurrence as a function of normalized Rabi frequencies ${\varOmega }_{1}/\gamma$ and ${\varOmega }_{2}/\gamma$ corresponding to two dipoles embedded inside the MNZ metamaterial and separated by **a** $0.5{\lambda }_{0}$, **b** ${\lambda }_{0}$, **c** $1.5{\lambda }_{0}$, **d** $5{\lambda }_{0}$, **e** $10{\lambda }_{0}$, and **f** $15{\lambda }_{0}$, assuming a normalized time of $\gamma t=90$ for the steady-state^20^. The coupling parameters used are given in Fig. 5a

Figure 6 shows the steady-state concurrence ${C}_{{{\rm {ss}}}}$ as a function of normalized Rabi frequencies ${\varOmega }_{1}/\gamma$ and ${\varOmega }_{2}/\gamma$ for the two quantum emitters embedded inside the MNZ metamaterial and separated by different distances *r*_12_ ranging from $0.5{\lambda }_{0}$ (Fig. 6a) to $15{\lambda }_{0}$ (Fig. 6f). The maximum steady-state concurrence [(*C*_ss_)] for each separation occurs at an antisymmetric pumping configuration of ${\varOmega }_{1}=-{\varOmega }_{2}$, except for $15{\lambda }_{0}$ where the (*C*_ss_) occurs at an asymmetric configuration. A similar analysis of bipartite entanglement between two quantum emitters placed inside a rolled-up ENZ waveguide has been previously analyzed^23^. It is interesting to note some important differences in the concurrence results between such an ENZ waveguide and the proposed MNZ metamaterial. First, the $\left({C}_{{{\rm {ss}}}}\right)$ values for both cases are comparable when ${r}_{12}=0.5{\lambda }_{0}$ (i.e., the emitters are within less than ${\lambda }_{0}$), with only slightly lower $\left({C}_{{{\rm {ss}}}}\right)$ for the MNZ metamaterial. However, at longer distances, such as ${r}_{12}={\lambda }_{0},\,{1.5\lambda }_{0},\,{5\lambda }_{0},\,{10\lambda }_{0},\,{15\lambda }_{0}$ the achievable $\left({C}_{{{\rm {ss}}}}\right)$ values for the MNZ metamaterial, surpass those of the ENZ waveguide. Remarkably, the $\left({C}_{{{\rm {ss}}}}\right)$ value achievable in the ENZ waveguide, even at a short dipole–dipole separation of ${r}_{12}{=1.5\lambda }_{0}$ falls short of the MNZ metamaterial with a large separation of ${r}_{12}=15{\lambda }_{0}$ (i.e., once again one order of magnitude enhancement in the entanglement range). This further confirms that the proposed MNZ metamaterial is far superior to the ENZ waveguide in mediating an entanglement between two quantum emitters over a long range—much longer than the free-space wavelength.

**Fig. 7:**
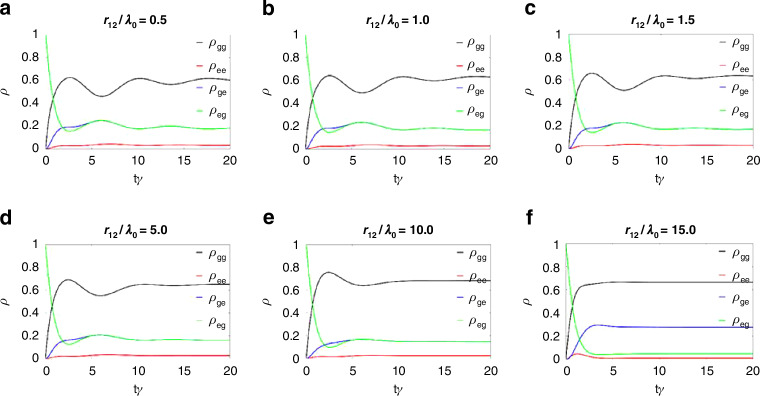
Time evolution. Time evolution of the diagonal elements of the density matrix at different dipole–dipole separations $r$ of **a** $0.5{\lambda }_{0}$, **b** ${\lambda }_{0}$, **c** $1.5{\lambda }_{0}$, **d** $5{\lambda }_{0}$, **e** $10{\lambda }_{0}$, and **f** $15{\lambda }_{0}$ inside the MNZ metamaterial, evaluated at the maximum concurrence values for each separation

Figure 7 displays the time evolution of the probabilities for the basis states (i.e., diagonal elements of the density matrix) using the same dipole–dipole separations as in Fig. 6. They are obtained at the Rabi frequencies (${\varOmega }_{1}$ and ${\varOmega }_{2}$) which give maximum concurrence for each dipole–dipole separation. Clearly, starting from the initial condition ${\rho }_{{{\rm {eg}}}}=1$, all the density matrix elements tend to reach the steady state at $\gamma t=20$, consistent with the steady-state assumption in Fig. 6. Towards the steady state, we observe the following changes in the density matrix elements with increasing separations between the dipoles from $0.5{\lambda }_{0}$ (Fig. 7a) to $15{\lambda }_{0}$ (Fig. 7f) or equivalently with the decreasing ${C}_{{{\rm {ss}}}}$. First, the probability ${\rho }_{{gg}}$ of finding both qubits in the ground state slightly increases, while the probability ${\rho }_{{{\rm {ee}}}}$ of finding both in the excited state slightly decreases. Second, although the single-excitation probabilities ${\rho }_{{{\rm {ge}}}}$ and ${\rho }_{{{\rm {eg}}}}$ overlap in Fig. 7a–e, they are also slightly reduced with the decreased ${C}_{{{\rm {ss}}}}$, and then dissociate in Fig. 7f, where the separation is $15{\lambda }_{0}$.

With the steady state density matrix of the present system, we can calculate the experimentally relevant photon–photon intensity correlations at zero time delay ${g}_{12}^{(2)}(0)$ as^39^

13 $${g}_{12}^{\left(2\right)}\left(0\right)=\frac{{{\langle }}{\sigma }_{1}^{\dagger }{\sigma }_{2}^{\dagger }{\sigma }_{2}{\sigma }_{1}{{\rangle }}}{{{\langle }}{\sigma }_{1}^{\dagger }{\sigma }_{1}{{\rangle }}{{\langle }}{\sigma }_{2}^{\dagger }{\sigma }_{2}{{\rangle }}}=\frac{{\rho }_{{ee}}}{({\rho }_{{eg}}+{\rho }_{{ee}})({\rho }_{{ge}}+{\rho }_{{ee}})}$$

**Fig. 8:**
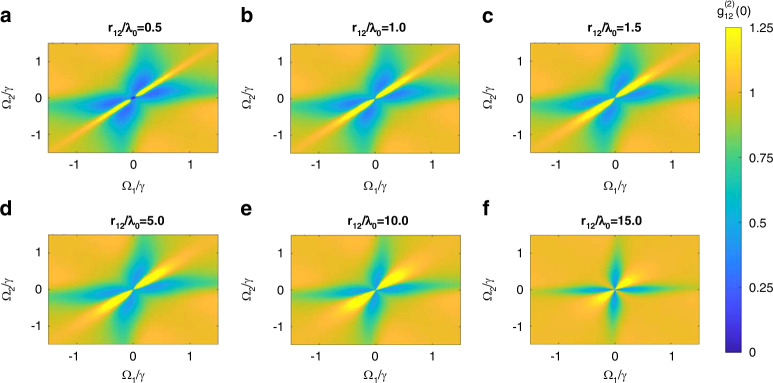
Steady-state zero time delay second-order correlation function. Steady-state zero time delay second-order correlation function ${g}_{12}^{(2)}(0)$ as a function of normalized Rabi frequencies ${\varOmega }_{1}/\gamma$ and ${\varOmega }_{2}/\gamma$ corresponding to the two dipoles in Fig. 6 separated by **a** $0.5{\lambda }_{0}$, **b** ${\lambda }_{0}$, **c** $1.5{\lambda }_{0}$, **d** $5{\lambda }_{0}$, **e** $10{\lambda }_{0}$, and **f** $15{\lambda }_{0}$

For the case of a plasmonic ENZ waveguide^21^, it has been demonstrated that the value of the zero time delay second-order correlation function ${g}_{12}^{(2)}(0)$ for two-level systems can transition from bunching to antibunching [${g}_{12}^{\left(2\right)}\left(0\right)=0]$ depending on the value of the ${\varOmega }_{1}$ and ${\varOmega }_{2}$, and across varying interdipole separations. The antibunching signature has been associated with a high degree of entanglement^39^. In Fig. 8, using Eq. (13), we calculate the photon–photon correlations for the two two-level systems in the diamond MNZ metamaterial corresponding to Fig. 6. We observe that the regions of higher concurrence (shades of orange in Fig. 6) generally tend to correlate with the smaller values of ${g}_{12}^{(2)}(0)$ (shades of blue in Fig. 8). However, the concurrence for the present system better captures the degree of entanglement than ${g}_{12}^{(2)}(0)$. These findings are consistent with the literature^21^. We also note that with the increasing separation between the quantum emitters, the parameter space for antibunching shrinks as expected, consistent with Fig. 6. All materials for this section are available online^53^.

### Considerations for experimental implementation

The above discussion is purely theoretical, including both analytical and numerical developments. However, our work stands as close as possible to experiments. Therefore, we would like to briefly discuss the potential of this theoretical work for experimental realization by discussing fabrication, characterization, and potential imperfections in the system.

Many solid-state quantum emitters experience spectral inhomogeneity in their optical transitions. This is generally due to local strain variations in the diamond induced via fabrication^54^. A way to work around this effect is by using a Raman transition. A common technique in atomic, molecular, and optical physics is to induce a stimulated Raman transition between two metastable states, also known as a two-photon transition^55^. The spectra of these transitions contain two components—one broad peak corresponding to spontaneous emission at frequency *ν* and a narrow Raman peak corresponding to *ν*−Δ, where Δ is the frequency of the detuning. For relatively large values of Δ, we can use this narrow Raman peak to represent the transition we are interested in.

It is possible to pump the device out-of-plane with a Ti:Sapphire laser detuned both above (the “dressing” laser) and below (the “pump” laser) 737 nm to excite a Raman transition near the ZPL in the SiV and measure the emission in-plane via a tapered waveguide. To measure the cooperative decay rate Γ, one can record the temporal profiles of either forward or backward atomic emission in the MNZ mode.

One can scan a Ti:Sapphire laser over a 700 GHz range centered around 406.8 THz at steps of around 100 MHz. One can record fluorescence counts in the phonon sideband around the ZPL as a function of frequency to acquire all emitter resonances in the device. SiV centers can be ionized from the SiV state to either the SiV0 or SiV+2 charge states. If using the Raman transition in the far detuned scheme, the latter two states are “dark” and do not emit^56^. To correct for ionization, one can apply a 532 nm laser pulse on the order of microseconds. This is called the “regenerating laser” in the setup. It returns the centers back to the SiV state.

**Fig. 9:**
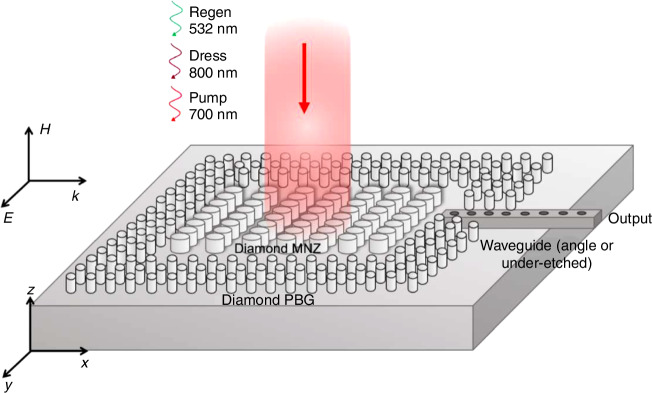
About experimental implementation. Schematic of MNZ material and PBG on the sides, with pumping out of plane and measuring in-plane with a waveguide

Figure 9 shows a simplified schematic of this measurement. For the output waveguide, it is possible to either use an angle-etched diamond waveguide or a diamond waveguide suspended by etching from underneath the substrate. Additionally, MNZ and photonic bandgap designs are both possible with airhole configurations in diamond, and thus the entire structure. It will likely be necessary to generate the short pump pulse (about 10 ns FWHM) to be able to fully decipher the enhanced decay rate.

Traditional fabrication in single-crystal bulk diamond is a challenging process that introduces specific requirements when both the nanostructures and the bulk are the same material. For example, etching waveguides onto bulk diamond requires angle-etching of the sides of the waveguides to prevent additional leakage into the substrate^56^.

Fortunately, recent research developments in diamond thin films have significantly improved the ease of fabrication and experimental feasibility of nanophotonic platforms with color vacancy centers in diamonds. For example, recent developments in fabricating nanophotonic devices and photonic crystals from thin-film diamond on silicon substrates show high yields and use conventional planar fabrication techniques^57^.

There are additional benefits to near-zero refractive index materials afforded to us due to the relaxed phase matching conditions, which allow spatial uncertainties in the implantation of the vacancy centers in the diamond pillars. In the plot of the effective refractive index in Fig. 2c, there is an approximately 20 nm region in which the effective refractive index of the MNZ material is at a minimum near zero, and throughout the entire simulation region of 100 nm, the absolute effective refractive index is <0.5. As Maxwell’s equations scale linearly with wavelength, small linear deviations of both pitch and radius will shift the wavelength of the effective constitutive parameters. While fabrication uncertainties (improper dosing of charge in electron beam lithography, under/over ion etching of devices) are not necessarily linear, small deviations in the pitch and radius of the photonic crystal within the range of near-zero refractive index behavior will not cause significant alterations in the effective refractive index.

**Fig. 10:**
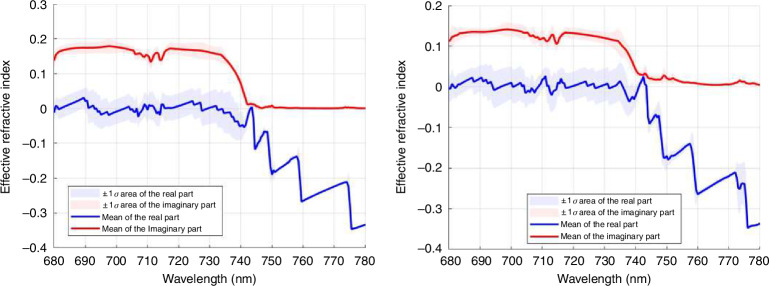
Effective refractive index of a 2D square lattice of diamond pillars (*n* = 2.4064) with a radius centered around 115 nm and randomly varied within ±2 nm (left) and ±5 nm (right). The crystal consists of 11 unit cells with a period $a=505\,{\rm {{nm}}}$, demonstrating near-zero-index behavior around 737 nm

To further demonstrate the relative robustness of the MNZ material design, we performed a simulation using COMSOL Multiphysics of our metamaterial with a pitch and radius of 505 and 115 nm, respectively, subjected to random perturbations of 2 and 5 nm on the cylinder radius (Fig. 10). These simulations demonstrate that under these perturbations, we still maintain an effective refractive index close to zero around 737 nm wavelength.

The proposed scheme could also be translated to other quantum emitters and technologies such as quantum dots, quantum wells, perovskite emitters, atoms, and cavity QED systems. Similar architectures have been previously proposed^31,32,58–63^ and the quantum master equation can still describe those systems. Furthermore, a key feature of the NZRI structures, as well as general photonic crystals and metamaterials, is their scalability in wavelength due to the scalability of Maxwell’s equations. Silicon vacancy centers in diamond here are chosen as candidate quantum emitters due to considerable experimental interest in them for the past decade; however, the simulation and design process to generate NZRI photonic crystals and metamaterials is easily generalizable for different materials and wavelengths. There are several candidate emitters that could be immediately used in place of silicon vacancy centers in diamond. Some examples include silicon zero-index metamaterials with quantum dots, or erbium-doped silicon^59^, which has narrow optical transitions between 1535 and 1538 nm.

## Discussion

We explored the dynamics of quantum entanglement within an MNZ metamaterial structure, emphasizing the temporal and spatial characteristics of concurrence. Our findings demonstrate that the MNZ structure significantly enhances entanglement between two quantum emitters beyond the capabilities of plasmonic ENZ waveguides and 2D all-dielectric ENZ platforms.

First, the concurrence maintained within the MNZ structure shows a robust enhancement, sustaining high levels of entanglement over distances up to 17 free-space wavelengths or ~12.5 µm. This is a substantial improvement—one order of magnitude—compared to both vacuum systems and ENZ waveguides.

More precisely, compared to free space, where cooperative decay enhancement significantly drops outside the range of a spatial wavelength to approximately the single emitter decay rate, with our MNZ material, we observe nearly a 16.5-fold spatial enhancement in the transient concurrence before we converge to the free space concurrence value. Extrapolating the results presented with ENZ plasmonic waveguide channels in ref. ^23^ to regions extending further than one micron, we observe a 2.96-fold enhancement in both cooperative enhancement and steady-state concurrence. Compared to the rectangular ENZ waveguides in ref. ^21^, we observe an 8.25-fold spatial enhancement in both the transient and steady-state concurrence as we increase the inter-emitter separation.

We should emphasize that the main scope of this work is how far apart two quantum emitters can be placed and yet entangle through the mediating medium. Typically, this is achieved either at very high proximity or via a prior interaction between the particles. Here, we show a significant increase in the entanglement range of quantum emitters on a chip. Photon pairs generated through four-wave mixing or spontaneous parametric down conversion can enable transmission over much longer ranges, and entanglement with high fidelity over the longest possible distance is desired to extend the practical range of quantum communication and cryptography systems^11,64^. However, such photonic entanglement or quantum communication should not be confused with the present scope. Nonetheless, one can cite some potential benefits of the proposed system on quantum computing and communications.

First, photonic entanglement over long distances is typically limited by losses and decoherence in the transmission medium^11,64^. Although the distance can be extended through entanglement purification, the overhead for this process exponentially grows with propagation distance^11^. In contrast, entangled quantum emitters on chip may allow for the construction of quantum repeaters for extended quantum communication distance with reduced overhead^11–13^. Second, preserving the high degree of entanglement on chip over such a distance may raise the possibility of multipartite entanglement^15,65,66^ involving many qubits, useful for e.g., the construction of cluster states as well as large-area distributed quantum computing and quantum communication networks that may offer a drastic increase in the computational and channel capacity. Multipartite entanglement has been investigated in 1D ENZ plasmonic waveguide systems^66^. 2D metamaterials like those proposed here would be of great interest, especially due to the additional dimension to separate the emitters.

Through the application of an external pump source, we extended the transient concurrence into a steady state, allowing for prolonged entanglement. The analysis reveals that the maximum steady-state concurrence is achieved under antisymmetric pumping configurations, except at the largest separation distances, where asymmetric configurations prevail.

Our comparative analysis further highlights that the MNZ metamaterial consistently surpasses the ENZ waveguide in terms of the maximum achievable concurrence across all studied separations. Even at shorter separations, where MNZ had only slightly lower concurrence, the longer-range performance proves far superior, reinforcing the MNZ structure’s efficacy in mediating long-range entanglement.

Finally, the time evolution of the system’s density matrix elements towards the steady state supports our conclusions. The probabilities of the ground and excited states adjust in accordance with the increasing separation between dipoles, reflecting the decreasing concurrence. The value of the zero time delay second-order correlation function ${g}_{12}^{(2)}(0)$ indicates an antibunching signature correlated with the high degree of concurrence.

In summary, the MNZ metamaterial offers a highly effective medium for sustaining quantum entanglement over extended distances and time periods. These findings pave the way for future innovations in quantum communication and computation, leveraging the unique properties of MNZ structures to facilitate robust and long-range quantum entanglement.

## Materials and methods

All calculations have been performed using Comsol Multiphysics and a homemade code. The homemade code is available on https://github.com/srnelson-mtu/MNZ-long-range-entanglement.

## Supplementary information

Supplementary information: Long-range quantum entanglement in dielectric mu-near-zero metamaterials

## Data Availability

The authors declare that all data supporting the findings of this study can be found within the paper and its Supplementary information files. Additional data supporting the findings of this study are available from the corresponding author (M.L.) upon reasonable request.
