# Supplementary material for: Long-range quantum entanglement in dielectric mu-near-zero metamaterials
Source: Light Sci Appl. 2025 Sep 3;14:300. doi: 10.1038/s41377-025-01994-9 (PMC12408817; doi:10.1038/s41377-025-01994-9)
Supplement: Supplementary file 1 — Supplementary information: Long-range quantum entanglement in dielectric mu-near-zero metamaterials [file 41377_2025_1994_MOESM1_ESM.pdf]

Supplementary information: Long-range quantum entanglement in dielectric mu-near-zero metamaterials

Olivia Mello[1], Larissa Vertchenko[2,] , Seth Nelson[3], Adrien Debacq[4], Durdu Guney[1,5], Eric Mazur[1], Michaël Lobet[1,4,*]

[1]John A. Paulson School of Engineering and Applied Sciences, Harvard University, 9 Oxford Street, Cambridge, MA 02138, USA
[2] Sparrow Quantum, 2100 Copenhagen, Denmark
[3]Physics Department, Michigan Technological University, Houghton, Michigan, USA
[4] Department of Physics and Namur Institute of Structured Materials, University of Namur, Rue de Bruxelles 51, 5000 Namur, Belgium
[5] Electrical & Computer Engineering Department, Michigan Technological University, Houghton, Michigan, USA

*Corresponding author: michael.lobet@unamur.be

1. Imaginary parts of effective permittivity, permeability and effective index

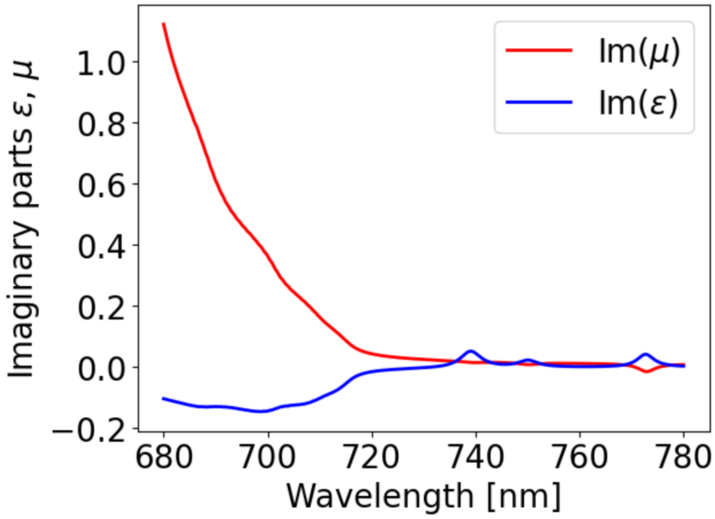

Figure S1. Imaginary parts of the effective permittivity and permeability corresponding to figure 2.

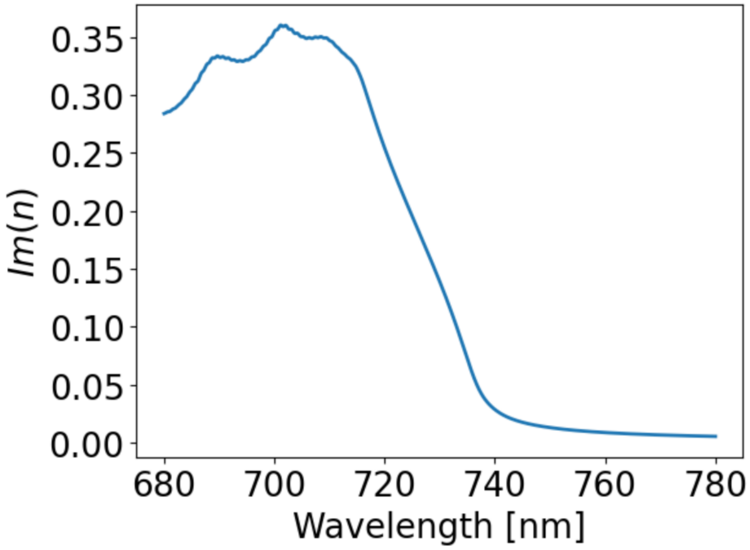

Figure S2. Imaginary parts of the effective refractive index corresponding to figure 2.

To double-check the consistency of the retrieved parameters using the parameter retrieval methods, we calculated the effective index n_{eff} calculated by averaging the phase advance between each pillar for 10 periods of the metamaterial using Ansys Lumerical FDTD (Figure S3). This agreement with $Re(\pm\sqrt{\varepsilon_r\mu_r})$ confirms the validity of the photonic crystal as a MNZ metamaterial
structure.

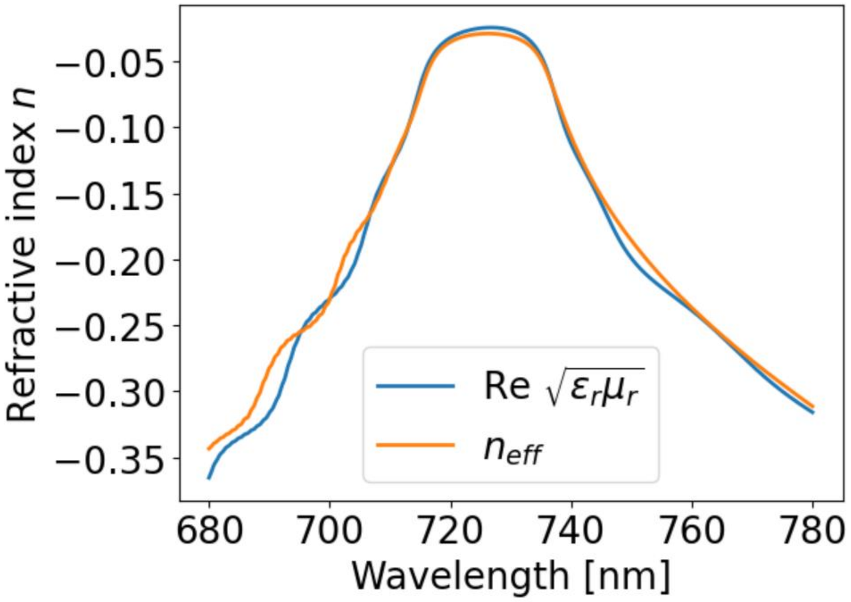

Figure S3. Comparison between the real part of the retrieved index $Re(\pm\sqrt{\varepsilon_r\mu_r})$ and the effective index n_{eff} calculated using FDTD.

2. Master Equations for the Quantum Dynamics of the Emitters in the Presence of Pump Lasers

The time evolution of the density matrix $\rho(t)$ in the presence of potential term (Eq. 12 in the main text) is described by the following set of 16 equations using the standard basis of $|0\rangle = |g1, g2\rangle, |1\rangle = |g1, e2\rangle, |2\rangle = |e1, g2\rangle$ and $|3\rangle = |e1, e2\rangle$, and assuming a zero detuning $\Delta_i = 0$.

$$\frac{\partial \rho_{00}}{\partial t} = i\Omega_1(\rho_{20} - \rho_{02}) + i\Omega_2(\rho_{10} - \rho_{01}) + \gamma_{11}(\rho_{22} + \rho_{11}) + \gamma_{12}(\rho_{12} + \rho_{21})$$

$$\frac{\partial \rho_{01}}{\partial t} = i\Omega_1(\rho_{21} - \rho_{03}) + i\Omega_2(\rho_{11} - \rho_{00}) + \gamma_{11}\left(\rho_{23} - \frac{1}{2}\rho_{01}\right) + \gamma_{12}\left(\rho_{13} - \frac{1}{2}\rho_{02}\right) - ig_{12}\rho_{02}$$

$$\frac{\partial \rho_{02}}{\partial t} = i\Omega_1(\rho_{22} - \rho_{00}) + i\Omega_2(\rho_{12} - \rho_{03}) + \gamma_{11}\left(\rho_{13} - \frac{1}{2}\rho_{02}\right) + \gamma_{12}\left(\rho_{23} - \frac{1}{2}\rho_{01}\right) - ig_{12}\rho_{01}$$

$$\frac{\partial \rho_{03}}{\partial t} = i\Omega_1(\rho_{23} - \rho_{01}) + i\Omega_2(\rho_{13} - \rho_{02}) - \rho_{03}\gamma_{11}$$

$$\frac{\partial \rho_{10}}{\partial t} = i\Omega_1(\rho_{30} - \rho_{12}) + i\Omega_2(\rho_{00} - \rho_{11}) + \gamma_{11}\left(\rho_{32} - \frac{1}{2}\rho_{10}\right) + \gamma_{12}\left(\rho_{31} - \frac{1}{2}\rho_{20}\right) + ig_{12}\rho_{20}$$

$$\frac{\partial \rho_{11}}{\partial t} = i\Omega_1(\rho_{31} - \rho_{13}) - i\Omega_2(\rho_{10} - \rho_{01}) + \gamma_{11}(\rho_{33} - \rho_{11}) - \frac{1}{2}\gamma_{12}(\rho_{12} + \rho_{21}) + ig_{12}(\rho_{21} - \rho_{12})$$

$$\frac{\partial \rho_{12}}{\partial t} = i\Omega_1(\rho_{32} - \rho_{10}) + i\Omega_2(\rho_{02} - \rho_{13}) - \rho_{12}\gamma_{11} + \gamma_{12}\left(\rho_{33} - \frac{1}{2}(\rho_{11} + \rho_{22})\right) + ig_{12}(\rho_{22} - \rho_{11})$$

$$\frac{\partial \rho_{13}}{\partial t} = i\Omega_1(\rho_{33} - \rho_{11}) - i\Omega_2(\rho_{12} - \rho_{03}) - \frac{3}{2}\rho_{13}\gamma_{11} + \rho_{23}\left(ig_{12} - \frac{1}{2}\gamma_{12}\right)$$

$$\frac{\partial \rho_{20}}{\partial t} = i\Omega_1(\rho_{00} - \rho_{22}) + i\Omega_2(\rho_{30} - \rho_{21}) + \gamma_{11}\left(\rho_{31} - \frac{1}{2}\rho_{20}\right) + \gamma_{12}\left(\rho_{32} - \frac{1}{2}\rho_{10}\right) + ig_{12}\rho_{10}$$

$$\frac{\partial \rho_{21}}{\partial t} = i\Omega_1(\rho_{01} - \rho_{23}) + i\Omega_2(\rho_{31} - \rho_{20}) - \rho_{21}\gamma_{11} + \gamma_{12}\left(\rho_{33} - \frac{1}{2}(\rho_{11} + \rho_{22})\right) + ig_{12}(\rho_{11} - \rho_{22})$$

$$\frac{\partial \rho_{22}}{\partial t} = -i\Omega_1(\rho_{20} - \rho_{02}) + i\Omega_2(\rho_{32} - \rho_{23}) + \gamma_{11}(\rho_{33} - \rho_{22}) - \frac{1}{2}\gamma_{12}(\rho_{12} + \rho_{21}) + ig_{12}(\rho_{12} - \rho_{21})$$

$$\frac{\partial \rho_{23}}{\partial t} = -i\Omega_1(\rho_{21} - \rho_{03}) + i\Omega_2(\rho_{33} - \rho_{22}) - \frac{3}{2}\rho_{23}\gamma_{11} + \rho_{13}(ig_{12} - \frac{1}{2}\gamma_{12})$$

$$\frac{\partial \rho_{30}}{\partial t} = -i\Omega_1(\rho_{32} - \rho_{10}) - i\Omega_2(\rho_{31} - \rho_{20}) - \rho_{30}\gamma_{11}$$

$$\frac{\partial \rho_{31}}{\partial t} = i\Omega_1(\rho_{11} - \rho_{33}) - i\Omega_2(\rho_{30} - \rho_{21}) - \frac{3}{2}\rho_{31}\gamma_{11} - \rho_{32}\left(\frac{1}{2}\gamma_{12} + ig_{12}\right)$$

$$\frac{\partial \rho_{32}}{\partial t} = -i\Omega_1(\rho_{30} - \rho_{12}) + i\Omega_2(\rho_{22} - \rho_{33}) - \frac{3}{2}\rho_{32}\gamma_{11} - \rho_{31}\left(\frac{1}{2}\gamma_{12} + ig_{12}\right)$$

$$\frac{\partial \rho_{33}}{\partial t} = -i\Omega_1(\rho_{31} - \rho_{13}) - i\Omega_2(\rho_{32} - \rho_{23}) - 2\rho_{33}\gamma_{11}$$

Here γ_{11} is the single qubit decay rate and γ_{12} is the cooperative decay rate. As the density matrix is Hermitian, the terms $\rho_{ij}(t)^* = \rho_{ji}(t)$. To solve for the steady state behavior of the system one can set the system $\partial\rho/\partial t = 0$ and solve for the eigenvalues of the matrix. Solving this secular equation gives us the steady-state density matrix elements ρ_{SS} and the steady-state concurrence C_{SS}. Alternatively, the steady-state results can be approximated by letting the system evolve over a sufficiently long-time duration (e.g., $\gamma t = 90$ as in Fig. 6 in the main text, see also Fig. 7).

Refs. 8 and 24 of the main text use the quantum master equation and provide some limited numerical results for two emitters in plasmonic waveguides under one or two pumps leading to steady-states (e.g., concurrence as a function of time under a single pump with varying Rabi frequencies or two pumps with a few specific configurations of the Rabi frequencies, and steady-state concurrence for a short range with a few specific configurations of the Rabi frequencies). Although more detailed numerical analyses of the steady-state concurrence and zero-time delay second order correlation function were provided in Refs. 21 and 23 of the main text, they fall short of providing the general coupled differential equations above for the density matrix

elements of the system in the presence of pumps to describe the full quantum dynamics of the two-qubit system under different initial conditions. Here we included those equations alongside a simplified analytical expression for the zero-time-delay second order correlation function in Eq. 13 in the main text as an entanglement measure relevant to experiments. Since the above equations are general in that they do not consider specific initial conditions, they can be directly employed to implement various quantum tasks.

3. Discussion about the advantage of MNZ vs ENZ vs EMNZ for coupling

The goal of this annex is to clarify the advantage of MNZ design compared to ENZ or EMNZ designs for coupling radiation into the system.

First, coupling is only possible along normal direction because of Snell's law of refraction. Indeed, if $n_2 = 0$ for the near-zero refractive index media to couple in, the only way to satisfy $n_1 sin\theta_1 = n_2 sin\theta_2$ from an incident medium different from a NZI ($n_1 \neq 0$) is to come at normal incidence ($\theta_1 = 0°$).

As we show below, it implies that the wave-vector must be zero for a plane wave propagating in a metamaterial with MNZ property.

From Maxwell's equations, this arises from the sourceless divergence of the electric field, which implies that the wavevector $\vec{k}$ must be orthogonal to the electric field $\vec{E}$:

$$\vec{\nabla} \cdot \vec{E} = 0 \implies \vec{k} \cdot \vec{E} = 0$$

Furthermore, since the permeability is zero in an MNZ medium, the electric field becomes irrotational, leading to:

$$\vec{\nabla} \times \vec{E} = 0 \implies \vec{k} \times \vec{E} = 0.$$

Thus, the only way the wavevector can be both parallel and orthogonal to the electric field is if the wavevector $\vec{k}$ is zero.

To couple with the zero-wavevector of a 2D photonic crystal, two coupling possibilities exist. First, in-plane coupling follows the wavevector defined at the Γ-point, aligning with the direction of periodicity (see Opt. Express 31, 26565-26576 (2023)[1]). Second, out-of-plane coupling involves a wave propagating perpendicular to the periodicity. If the periodicity is in the *xy*-plane, a wave propagating along the *z*-direction results in a zero wavevector in the *xy*-plane (see Nature 608, 692–698 (2022)[2]).

MNZ design better couples light in over ENZ design[3] because of the diverging impedance $Z = \sqrt{\frac{\mu}{\varepsilon}}$ in the ENZ case[4]. Nevertheless, in the case of a 2D MNZ photonic crystal, in-plane coupling is weak due to the impedance approaching zero, which leads to high reflectance at the zero refractive index frequency. However, above the bandgap ($R \to 1$) but still in the near zero refractive index regime (e.g. between 740-760 nm), light is transmitted according to Fabry-Perot oscillation,

enabling in-plane coupling (Figure S4a). Further optimizations are required to maximize this coupling.

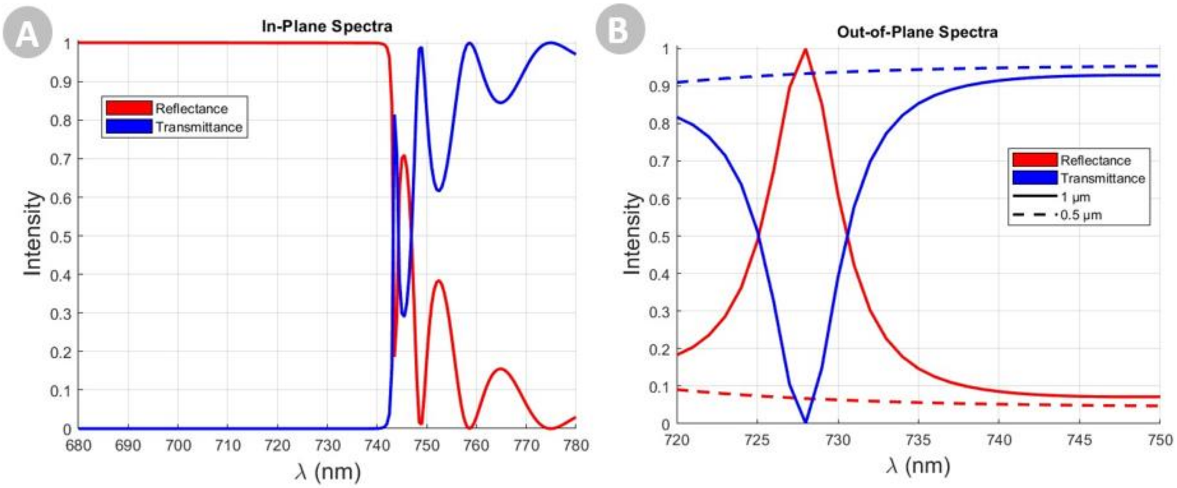

Figure S4 : (a) In-plane transmittance and reflectance spectrum of a 2D square lattice of diamond pillars ($n = 2.4064$, radius $r = 115$nm). The lattice consists of 11 unit cells with a period $a = 505\,nm$, showing MNZ behavior around a wavelength of 737 nm. (b) Out-of-plane transmittance and reflectance spectrum of a 2D square lattice of diamond pillars for different thicknesses. The lattice size is 5 unit cells with a period.

In contrast, out-of-plane coupling does not suffer from this Fabry-Perot oscillations since the MNZ behaviour is confined to the plane. The efficiency of out-of-plane coupling depends on the thickness of the crystal (Figure S4b) and can be optimized as described in Light Sci Appl 10, 10 (2021)[5].

In conclusion, coupling to the MNZ photonic crystal can occur both in-plane and out-of-plane.

Comparing to an EMNZ is a different problem. The impedance of an EMNZ structure responds according to a L'Hospital rule at the NZI limit and reaches $Z = \sqrt{\frac{\frac{d\mu}{d\omega}}{\frac{d\varepsilon}{d\omega}}}$ [4]. Therefore, a careful engineering of dispersion relation of both effective ε and μ since there could have their independent slope variation as we reproduced the results from [6] here below (Figure S5):

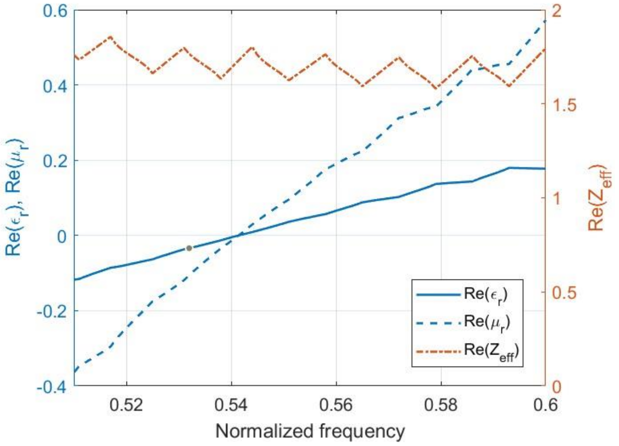

Figure S5 : Real permittivity and real permeability (left) reproduced from the structure simulated in [6], figure 2. It clearly shows that the slope of the permittivity and permeability can be different, leading to an effective impedance different from unity.

Nevertheless, EMNZ materials can be considered as a candidate for performance. The figure S6 below demonstrates the cooperative enhancement of a TM-polarized EMNZ material. We calculate this cooperative enhancement in the same manner as in Figure 5a of the manuscript, sweeping a line current dipole source across the high symmetry points of the EMNZ material while keeping another dipole source at the center of the material fixed. This metamaterial has a pitch of $a = 473\,nm$ and a pillar radius is $r = 113\,nm$. We calculate the cooperative enhancement by calculating the ratio of enhancement of the imaginary component of the electric field Ez for the two dipoles as they increase in separation to the field of the single dipole. This metamaterial offers a similar level of cooperative enhancement as the MNZ material in the manuscript.

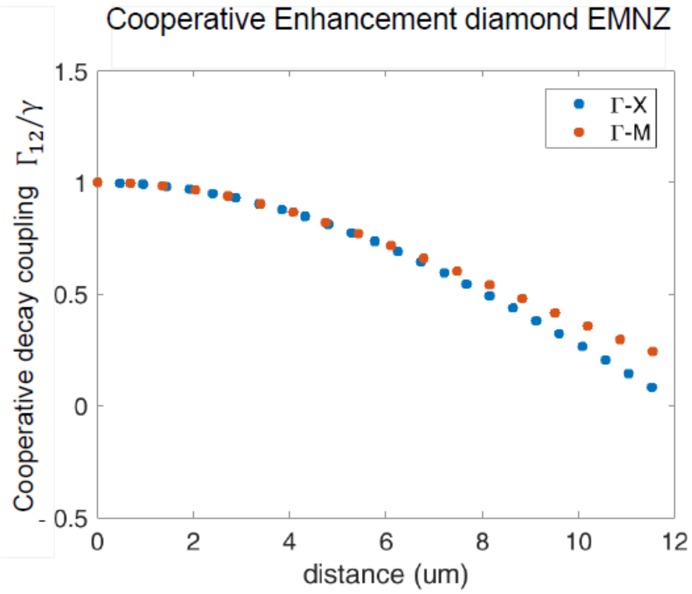

Figure S6 : The cooperative decay rate coupling Γ_{12}/γ for a diamond EMNZ metamaterial in both the $\Gamma - X$ and $\Gamma - M$ directions.

The main advantage of using ENZ (TM polarization) or MNZ (TE polarization) is due to the Purcell enhancement one receives when the triply-degenerate Dirac cone at the Gamma point of EMNZ materials is detuned into the parabolic dispersion at the Gamma point that we observe in ENZ and MNZ materials. This parabolic dispersion and resulting band edge at the Gamma point introduces a significant decrease in the group velocity of the light at that point, which in turn increases the local density of states and thus the Purcell factor. The figure below shows the Purcell enhancement we observe in the MNZ material at 737 nm (see Figure 3a of the manuscript).

While this does not affect the overall cooperative enhancement, this does generate a greater overall emitted power and signal. The additional broadening of the linewidth occurring due to emission at the bandedge of either an MNZ or ENZ material also helps increase the indistinguishability of individual silicon vacancy centers emitting near the zero-phonon line.

References

1. Zeng, Y., Han, S., Zheng, G., Li, Z. & Zeng, Y. In-plane emission manipulation of random optical modes by using a zero-index material. *Opt. Express* **31**, 26565 (2023).
2. Contractor, R. *et al.* Scalable single-mode surface-emitting laser via open-Dirac singularities. *Nature* **608**, 692–698 (2022).

3. Mello, O. *et al.* Extended many-body superradiance in diamond epsilon near-zero metamaterials. *Applied Physics Letters* **120**, 061105 (2022).
4. Lobet, M. *et al.* Fundamental Radiative Processes in Near-Zero-Index Media of Various Dimensionalities. *ACS Photonics* **7**, 1965–1970 (2020).
5. Dong, T. *et al.* Ultra-low-loss on-chip zero-index materials. *Light Sci Appl* **10**, 10 (2021).
6. Huang, X., Lai, Y., Hang, Z. H., Zheng, H. & Chan, C. T. Dirac cones induced by accidental degeneracy in photonic crystals and zero-refractive-index materials. *Nature Materials* **10**, 582–586 (2011).
